# Low-Dose Niacin Supplementation Improves Motor Function in US Veterans with Parkinson’s Disease: A Single-Center, Randomized, Placebo-Controlled Trial

**DOI:** 10.3390/biomedicines9121881

**Published:** 2021-12-10

**Authors:** Chandramohan Wakade, Raymond Chong, Marissa Seamon, Sharad Purohit, Banabihari Giri, John C. Morgan

**Affiliations:** 1 Charlie Norwood VA Medical Center, Augusta, GA 30904, USA; rchong@augusta.edu (R.C.); mseamon@augusta.edu (M.S.); spurohit@augusta.edu (S.P.); bgiri@augusta.edu (B.G.); jmorgan@augusta.edu (J.C.M.); 2 Center for Biotechnology and Genomic Medicine, Medical College of Georgia, Augusta University, Augusta, GA 30912, USA; 3 Department of Physical Therapy, College of Allied Health Sciences, Augusta University, Augusta, GA 30912, USA; 4 Department of Neuroscience and Regenerative Medicine, Medical College of Georgia, Augusta University, Augusta, GA 30912, USA; 5 Department of Neurology, Medical College of Georgia, Augusta University, Augusta, GA 30912, USA; 6 Department of Interdisciplinary Health Sciences, College of Allied Health Sciences, Augusta University, Augusta, GA 30912, USA; 7 Department of Undergraduate Health Professions, College of Allied Health Sciences, Augusta University, Augusta, GA 30912, USA

**Keywords:** Parkinson’s disease, movement disorder, niacin, UPDRS III, fatigue

## Abstract

A six-month double-blind, placebo-controlled randomized study was conducted to ascertain whether low-dose daily niacin supplementation would improve motor symptoms in Parkinson’s disease (PD) patients. A total of 47 PD patients were assigned to receive low-dose niacin or a placebo. At the end of the double-blind phase, all participants received open-label niacin for the next six months. All patients were evaluated at baseline, after six months, and after one year of treatment. The primary outcome measure was the Unified Parkinson’s Disease Rating Scale III (UPDRS III) scores. Secondary outcome measures were depression, sleep quality, mental flexibility and cognition, and physical fatigue. Niacin treatment was well-tolerated by forty-five subjects. The mean [95% CI] change in UPDRS III scores at six months of placebo was −0.05 [95% CI, −2.4 to 2.32], and niacin was −1.06 [95% CI, −3.68 to 1.57]. From six to twelve months when both groups received open-label niacin supplementation, the average UPDRS III scores significantly decreased for the placebo group by 4.58 [95% CI, −0.85 to 8.30] and the niacin group by 4.63 [95% CI, 1.42 to 7.83] points. Low-dose niacin supplementation is a well-tolerated adjunct therapy and may improve motor function in PD when taken over a longer period.

## 1. Introduction

Parkinson’s disease (PD) is one of the most common movement disorders afflicting approximately 1% of the population above the age of 60 and 4% by age 80. A definitive diagnosis for PD requires an autopsy, and there is no cure or definitive disease-slowing therapy despite extensive investigation [1]. Sinemet (carbidopa/levodopa) remains the cornerstone of PD therapy but can lead to significant motor complications (wearing off and dyskinesia) over time [2].

Numerous pathophysiological processes interplay in PD, but neuroinflammation and mitochondrial dysfunction remain at the core of PD pathology [3,4,5,6]. Niacin is, therefore, a promising choice as an adjunct therapy in PD since it is anti-inflammatory and boosts mitochondrial function by providing NAD [5,6]. Parkinson’s disease is accompanied by non-motor symptoms such as lack of sleep, depression, and fatigue, resulting in poor quality of life [7,8,9]. Medications that reduce motor symptoms further aggravate non-motor symptoms in PD patients [10]. Moreover, carbidopa in Sinemet is known to deplete niacin levels in treated PD patients [11]. Chong et al. recently investigated niacin in a three-month effectiveness trial, showing improvements in quality of life and motor symptoms in PD patients [12]. Our group and others have indicated that niacin supplementation may influence outcomes in PD patients [5,12,13].

The present study investigates the effects of low-dose niacin treatment and improvement in tremor, rigidity, and overall Unified Parkinson’s Disease Rating Scale III (UPDRS III) scores in US veterans with PD. Compared to the study by Chong et al. [12], this trial has a longer randomized, double-blind, placebo-controlled phase, followed by an additional six months of open-label niacin.

## 2. Materials and Methods

### 2.1. Participants

The study was a single-center prospective trial conducted at Charlie Norwood Veterans Affairs Medical Center (CNVAMC, GA, USA) neurology clinic and Augusta University Medical Center (AUMC, GA, USA) Tertiary Movement Disorders Center (clinicaltrials.gov identifier: NCT03462680). The patients were enrolled from October 5th, 2016 to September 18th, 2019, and tested every six months. The inclusion criteria were: (1) mild to moderately severe PD patients according to the United Kingdom Parkinson’s Disease Society Brain Bank Diagnostic Criteria [14], (2) Hoehn and Yahr (H&Y) scores between 0.5–4, (3) stabilized on all PD medications prior to enrollment with expected medication stability for at least six months, and (4) mini-mental state exam (MMSE) scores above 24. The exclusion criteria were: (1) other severe neurological problems, previous brain surgery, functional blindness, inability to participate in visuomotor or gait assessments, (2) an allergy to niacin, (3) other severe illnesses, (4) previously undergone deep brain stimulation, or (5) dementia. An attending expert neurologist used his clinical judgment to determine if a patient was suitable for the study, including an optional spinal tap. The study was conducted according to the Declaration of Helsinki (1997) and approved by The Institutional Review Board at AUMC (750415). Demographic, clinical, and medication data were captured from the patient database. All subjects signed a written informed consent to participate in the study.

### 2.2. Intervention

**Figure 1:**
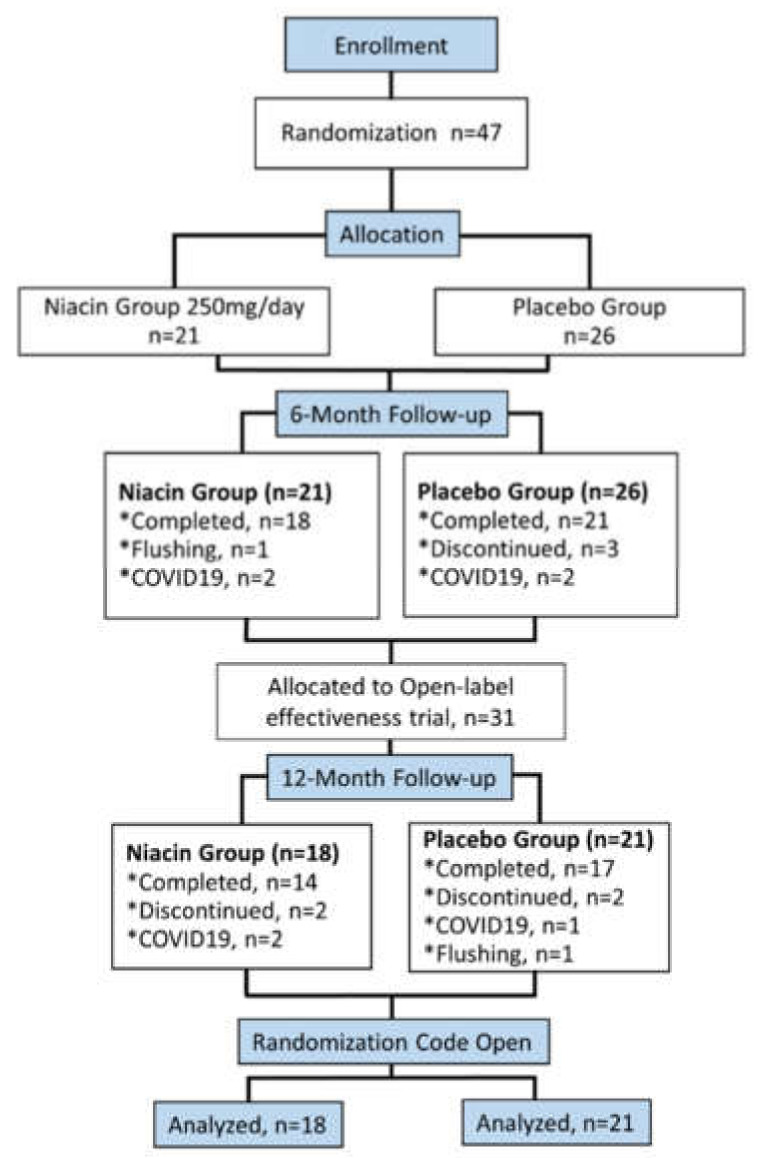
Flowchart showing participant flow in the Niacin for Parkinson’s disease trial. A total of 47 participants enrolled in the study were randomly placed into the placebo or niacin-treated group. The randomized, double-blind portion lasted six months; then, a subsequent open-label niacin phase was implemented from six to twelve months.

A total of 47 patients, stabilized on medications three months prior, were enrolled in the study. The subjects were kept on the same dose of medications for the first six months of the study. Patients were randomized and blinded to receive either 250 mg of niacin once daily or placebo in accordance with the sequestered fixed randomization schedule (Figure 1), using balanced blocks to ensure an approximate 1:1 ratio of the two treatment arms for early-stage (H&Y 0.5–2) or late-stage patients (H&Y 2.5–4). The VAMC pharmacy generated the randomized sequence for the group assignment. Study subjects and the researchers were blinded to the allocation. Grouping was also concealed by dispensing the supplements (placebo and niacin) in an opaque pouch. The randomization code was not revealed to the investigators until the study was completed in April 2020. The facility for performing outcome measures was provided by the CNVAMC.

### 2.3. Outcomes of the Study

The study’s primary outcome was a change in the UPDRS III scores from baseline to six months and one year. Based on pilot data, the margin of superiority representing the minimal clinically meaningful change in score, δ, is 5. This value is the expected median annual rate of decline in the UPDRS III score of +5.5 points [15,16]. Secondary outcomes included depression rating by the Geriatric Depression Scale (GDS), fatigue rating by the Fatigue Severity Scale (FSS) and Visual Analogue Fatigue Scale (VAFS), mental resilience measured by Trail Making Test (TMT) A and B (the difference between TMT-B and TMT-A was considered as a measurement of cognitive flexibility) [17], cognitive ability and mental fatigue through the Stroop Test, overall cognitive function by MMSE, amino acid and serotonin levels, and physical strength and fatigue through a grip strength test. A single assessor took all measurements to eliminate interrater differences. Patients were tested at baseline, after six months of daily 250 mg niacin or placebo, and then again after six months of open-label 250 mg daily niacin. There were no treatment changes (regarding medications and dosages) made during the double-blind trial period.

### 2.4. Statistical Analysis

The trial data was analyzed according to the intent to treat and per-protocol methods. Means, standard deviations or standard errors, and ranges were calculated for continuous and numerical data. Categorical data were presented as counts and percentages, analyzed by chi-sq test. Statistical differences were tested between the treatment groups and time points by ANOVA with Tukey’s post hoc corrections for primary outcomes. All *p*-values were adjusted for multiple comparisons according to Bonferroni’s correction. A *p* ≤ 0.05 was considered statistically significant; all *p* values were two-sided.

The sample size of thirty-nine analyzed patients was determined to give 80% power (α = 0.05) to detect a reduction of 2.5 points in UPDRS III scores after six months of treatment [15,16]. For a significance level (α) of 5% and a power (1-β) of 80%, an expected standard deviation of the difference between data pairs was 3.9, and the minimum sample size was 13 participants per group. Our study sample was substantially increased to take into account potential dropouts or unexpected increases in variability.

Data for age, sex, race, and PD medications and dose were documented from the patient records system. Laboratory data on serum levels of serotonin was also obtained. Data for all participants was entered into an Excel sheet (Microsoft Excel v2016) for subsequent analysis. Outcomes of all 39 participants who completed the six-month trial were analyzed according to their randomization groups, comparing differences between treatments and differences between time points of baseline, six months, and one year.

One outlier was removed from UPDRS III scores based on the ordinary least-squares regression test (Q = 10%). All other outcome data had no outliers removed by the robust regression and outlier removal test. All analyses were performed using Prism (v8.0, Graphpad, San Deigo, CA, USA).

## 3. Results

### 3.1. Subjects

Between 2016–2019, 47 participants were enrolled at the CNVAMC; 39 returned for the six-month evaluation. Participants were randomly assigned to the placebo (n = 21) or 250 mg daily niacin (n = 18) group (Figure 1). The mean age was 68.4, with a mean duration of PD being 5.8 years (Table 1). A total of 36 participants were Caucasian, and three were African American; four participants were women. Based on H&Y scores, the subjects were divided into early (n = 28) and late-stage (n = 11) groups. Veterans (n = 25) constituted 64 % of the total study subjects (n = 39), while remaining patients were non-veterans (n = 14) (Table 1).

A total of 35 subjects were on Sinemet as their primary Parkinson’s medication throughout the study. The neurologist changed the medication dose of five patients during the open-label phase of the study (Table 1). Other medications or supplements taken by subjects included Trihexyphenidyl (n = 2), Donepezil (n = 1), Simvastatin (n = 1), Diclofenac (n = 1), Temazepam (n = 1), Aspirin (n = 7), Ropinirole (n = 8), Rasagiline (n = 9), Pramipexole (n = 13), Amantadine (n = 1), Vitamin D3 (n = 2), multivitamin (n = 5), and Vitamin C (n = 1). None of the subjects in this study were on SSRIs.

The six-month follow-up was completed by 39 of the 47 enrolled patients. Of these, eight of the 39 patients did not complete the optional six-month open-label portion of the study (Figure 1). Participants discontinued due to the flushing effect of niacin (n = 2), voluntary discontinuation (n = 7), or the SARS-CoV-2 shut-down of clinical research (n = 7). Chi-squared and Fisher tests showed no significant differences in the dropout rate between the groups, *p* = 0.65 and 0.72, respectively. Although both groups took niacin from six to twelve months, the group who took the placebo for the first six months will continue to be referred to as the placebo group for this study (Figure 1).

### 3.2. Primary Outcome: UPDRS III

#### 3.2.1. Six Months of 250 mg Niacin vs. Placebo

**Figure 2:**
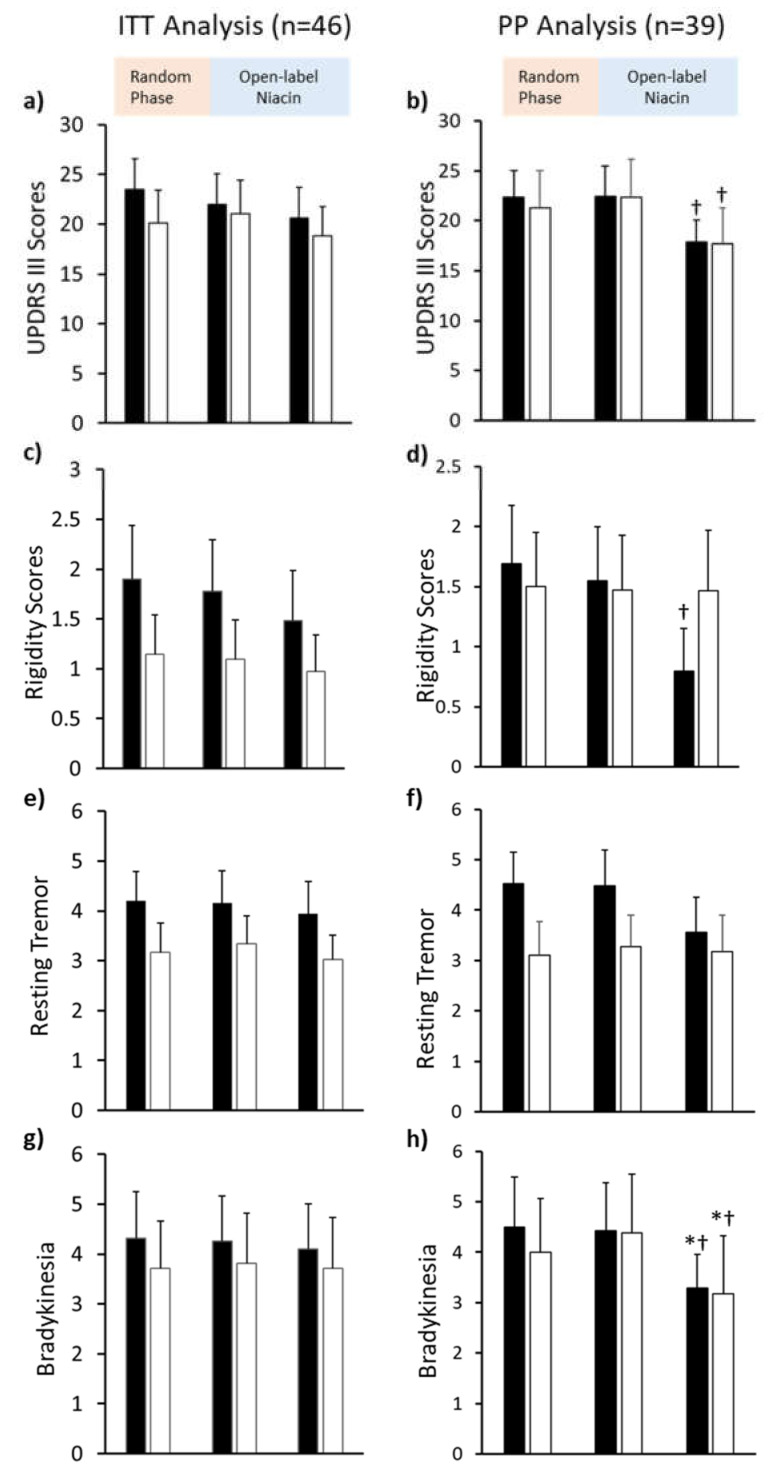
Mean Unified Parkinson’s Disease Rating Scale III (UPDRS III) scores for two treatment groups analyzed per-protocol (right) and intention-to-treat with last observation carried forward (left). (**a**,**b**) Bar plot comparing changes in UPDRS III scores at baseline, after six months (6MO) of placebo (grey bars) or niacin (open bars), and after six months of niacin thereafter (12MO) (maximum points: 108). (**c**,**d**) Bar plot for rigidity scores (maximum points: 20), as a component of the UPDRS III scores, (**e**,**f**) Bar plot showing resting tremor scores (maximum points: 20), as a component of UPDRS III scores, and (**g**,**h**) Bar plot showing for bradykinesia scores (maximum points: 20), as a component of UPDRS III scores. * 12-month compared to baseline *p* < 0.05, † 12-month compared to 6-month *p* < 0.05. Values presented are mean ± SEM.

In intention-to-treat (ITT) analysis, there were no significant differences (Figure 2a,c,e,f). We then analyzed the data on a per-protocol (PP) basis (Figure 2b,d,f,h). The mean UPDRS III scores at baseline for placebo (22.4 ± 11.8) and niacin (21.3 ± 15.8) groups were comparable (Figure 2b and Supplementary Table S1). The mean changes in UPDRS III scores at six months for niacin (−1.06 [−3.68 to 1.57]) and placebo (−0.05 [−2.4 to 2.32]) groups were not significant (Table 2). No significant changes were noted in rigidity, bradykinesia, or resting tremor in either placebo or niacin-treated subjects. However, non-significant differences in the mean scores between baseline and six months were observed in both groups for these variables (Table 2). Average scores for rigidity non-significantly decreased in the placebo (0.14 [−0.14 to 0.42]) and niacin (0.03 [−0.26 to 0.31]) groups. The average score for resting tremor changed by 0.05 [−0.54 to 0.64] in the placebo group and −0.17 [−0.49 to 0.16] in the niacin-treated group. Mean scores for bradykinesia changed by 0.07 [−0.35 to 0.49] in the placebo group and by −0.39 [−1.42 to 0.65] in the niacin group (Table 2).

#### 3.2.2. Six to Twelve Months of 250 mg Niacin

At the twelve-month visit, the mean UPDRS III scores significantly decreased by 4.58 [0.85 to 8.30] points in placebo compared to the six-month visit (Table 3). In the niacin group, the mean UPDRS III scores significantly decreased by 4.63 [1.42 to 7.83] points (Table 3). Bradykinesia scores were reduced in the placebo (1.13 [0.25 to 2.02]) and niacin (1.21 [0.36 to 2.06]) groups at the twelve-month visit compared to the six-month visit (Table 3). At the twelve-month visit, rigidity scores significantly decreased in the placebo group (0.75 [−0.01 to 1.51], *p* = 0.05), while no changes were observed in the niacin group (0.008 [−0.47 to 0.49]. No significant changes were observed in resting tremor scores for placebo (0.92 [−0.12 to 1.96]) or niacin (0.1 [−0.56 to 0.76]) groups.

#### 3.2.3. Baseline to Twelve Months

Compared to baseline, a trend towards reduction in the mean UPDRS III scores was observed in the placebo (4.52 [−0.16 to 9.21]) and niacin (3.57 [−1.02 to 8.16]) groups (Table 4). At twelve months, scores for rigidity (0.90 [−0.05 to 1.84]) and resting tremor (0.97 [−0.16 to 2.09]) showed slight, non-significant decreases in the placebo group. Compared to baseline, bradykinesia scores at twelve months significantly decreased in the placebo (1.21 [0.19 to 2.23]) and niacin (0.82 [−0.004 to 1.65]) groups (Table 4).

A significant decrease occurred across both groups over a treatment period of one year (*p* = 0.012), but no significant differences were found between treatments at any time point (Figure 2d and Table 4).

### 3.3. Correlation with on/off Phenomenon with UPDRS III Scores

**Figure 3:**
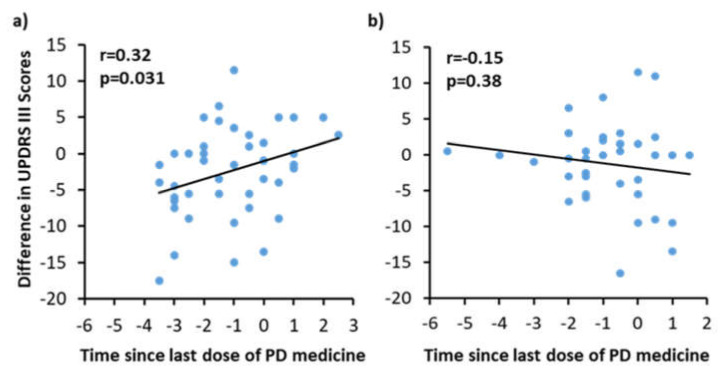
Correlation between time since last medication (*x*-axis) versus Unified Parkinson’s Disease Rating Scale III (UPDRS III) scores (*y*-axis) for the (**a**) placebo and (**b**) niacin groups. The UPDRS III scores and the change in the time between the last dose of medication and the appointment were used to calculate the difference between visits; this data was used to construct dot plots and run a correlation analysis.

The placebo group showed a positive correlation (*p* = 0.031) between UPDRS III scores and the time since their most recent dose of PD medication (on/off phenomenon) (Figure 3a). Subjects in the niacin group showed a negative correlation (r = −0.15), suggesting that the on/off phenomenon has no effect on UPDRS III scores in patients taking niacin (Figure 3b).

### 3.4. Secondary Outcomes

Grip strength, a measure of motor function and muscle energetics, showed a trending increase in the niacin group for each hand (22.56 [−53.54 to 8.43]); (31.67 [−63.74 to 0.39] PSI). However, those who took the placebo showed no change in either hand (5.88 [−14.19 to 25.94] PSI); (3.73 [−35.11 to 42.56] PSI) (Table 2). The difference between TMT-B and TMT-A is a measurement of cognitive flexibility while removing the factors of motor and visuoperceptual deficits. The change in cognitive flexibility between all the comparisons was not significant. Mean differences between the placebo and niacin groups changed from −6.04 to 22.52 over the study period (Table 2). Blood serotonin levels significantly decreased in placebo by 25.34 mg/dL [95% CI, 5.79 to 44.89] while staying relatively stable between baseline and six months of niacin supplementation (Table 2).

Rapid eye movement (REM) sleep changed significantly by 6.26% [−11.05 to −1.47] with placebo, but not with niacin (1.39% [−5.42 to 8.2]). FSS scores at twelve months decreased by 4.36 [1.59 to 7.13] points in the niacin-treated group (Table 3). No significant differences were observed for VAFS, GDS, or Stroop test (Table 2). Serum levels of amino acids valine, tyrosine, tryptophan, phenylalanine, leucine, and isoleucine were also not different between the treatment groups. Sleep efficiency or percentage of light sleep, deep sleep, or awake time did not significantly differ during the study (Table 2).

### 3.5. Adverse Events

Adverse events were similar between the niacin and placebo groups. Out of 47 recruited patients, eight patients dropped out during the first six months, and eight more dropped out before the one-year time point. The flushing effect of niacin occurred and caused discontinuation for two patients, one in each group. Unrelated injuries occurred in one patient in the niacin group and one in the placebo group, although neither resulted in the discontinuation of the study. One patient complained of leg cramps at the end of the study, but it was likely dehydration rather than supplement intake, assessed by the neurologist. No other adverse events were reported. Seven patients could not complete the study due to the SARS-CoV-2 shut-down.

## 4. Discussion

In this single-center, double-blind, placebo-controlled, randomized clinical trial comprising patients with early- and late-stage PD, supplementation of a single daily dose of niacin for twelve months improved the rigidity, bradykinesia, and overall UPDRS III scores. In a serendipitous finding, a high-dose niacin supplement was observed to reduce bradykinesia and rigidity in a PD case report [18]. Niacin supplements and agonists of the niacin receptor are shown to be neuroprotective and lead to enhanced motor function in animal models of PD (BHB, niacinamide, PINK1 fruit fly study) [19,20,21]. The current study demonstrated a significant decrease in UPDRS III scores from six to twelve months, in which both groups took the open-label niacin supplement. Both groups, when comparing baseline to one year, demonstrated a trend towards reduction in UPDRS III scores. There was a significant decrease in bradykinesia between baseline and twelve months and between six months and twelve months in both placebo and niacin-treated groups. A significant decrease in rigidity was observed in the placebo-treated group from six months to one year when they received niacin. Although the overall tremor score did not show any significance in the niacin-treated group, individual limb tremor scores decreased in the niacin-treated group. The placebo group showed a reduction in tremor scores from six months to twelve months after receiving niacin. Since some of the scores began higher (although insignificant) in the placebo group than niacin, such as with resting tremor, then there is more room for improvement; this may have contributed to the significant changes with only six months of niacin during the open-label portion for the placebo group opposed to the niacin group in the first six months. Taken together, small changes in these sub-scores reflect a decrease in UPDRS III scores at twelve months.

Numerous studies have attempted either raising NAD levels or directly providing NAD in PD. Niacin remains a natural source for NAD and binds to the niacin receptor, G protein-coupled receptor 109A (GPR109A), unlike other forms of vitamin B3. GPR109A is predominantly expressed in adipose tissue and immune cells, including macrophages [22]. We have previously shown that RAW264.7 macrophage cells stimulated with lipopolysaccharide express increased levels of GPR109A, and treatment with niacin reduces these levels [23]. Previously, niacin has been shown to have anti-inflammatory effects on activated macrophages in cardiovascular disease [24]. Animal and human evidence suggests that inflammation and activation of microglia in the brain are associated with the development and progression of PD [13,25,26]. Microglial cells in the brain are a modified form of macrophages that reside in the brain; we theorize that niacin supplement provides the anti-inflammatory response to these microglial cells to reduce the neuroinflammation in PD patients.

Figure 3 partially explains the effect of decreased UPDRS III scores in the placebo group during the first six months. Since Sinemet’s effects are only motor, this correlation likely does not affect data involving the non-motor abilities of the subjects enrolled in the clinical trial.

We have previously shown that niacin and NAD levels in PD patients are decreased compared to age-matched controls. Fatigue, depression, strength, sleep, and quality of life indicators may be affected by the reduction in niacin levels. In this study, six months or one year of niacin reduced typical PD motor symptoms and fatigue and stabilized serotonin levels and REM sleep percentage. These effects are highly beneficial for the PD patient’s quality of life. Many PD patients complain of both depression and fatigue. This study showed a halting or slowing of the drop in serotonin levels by niacin, which could potentially reduce depression or improve mood. Additionally, fatigue is likely enhanced by increasing NAD, which is low in aging populations and even lower in PD. Grip strength, although not significant, showed a trend of increasing strength bilaterally in the niacin group, but not with placebo over six months of treatment. Grip strength is well-known to correlate with longevity, so this could be a predictor of increased life expectancy by taking niacin [27]. Almost all reported secondary measures demonstrated a trend towards improvement over one year.

Most notably, serotonin levels, fatigue, and grip strength stood out in our study. Serotonin levels were significantly lowered in the placebo-treated group but not in the niacin-treated group after the first six months. The FSS scores were markedly better in the niacin-treated group but not the placebo group from six to twelve months. Both of these findings may explain the reporting of an uplift in mood reported by PD subjects who received niacin for one year.

To our knowledge, this is the first trial where niacin (a form of vitamin B3) is tested at a low dose for PD in a randomized, placebo-controlled, double-blind, prospective trial. A 250 mg daily dose of niacin produced negligible flushing symptoms when consumed after meals as instructed. Our previous preliminary studies helped us decide the low dose for niacin [12,13]. Different low-dose regimens, longer duration of intervention, multicenter trials, and inclusion of niacinamide would be pertinent future investigations.

### 4.1. Study Strengths

The commonly used therapies for PD subjects do not currently include niacin. It is a novel approach utilizing an over-the-counter vitamin supplement at a low dose in PD. A placebo control was used for the first six months, and codes were not broken until patients completed the twelve-month study. The flushing effect of niacin was negligible and did not deter the PD patients from the study. The PD patients and caregivers were very enthusiastic after the six-month open-label niacin supplementation. Dropout rates were low. A single rater captured the motor outcome scores for all of the subjects, which extended higher confidence in the reliability of the outcomes. Since PD is a slow, progressive disease, and thus, six months may not be enough to detect all meaningful changes in symptoms and may require longer durations, such as twelve months or longer interventions (9).

### 4.2. Study Limitations

The sample size is small. We did not titrate the dosages of niacin. The clinical trial recruited patients primarily through the Charlie Norwood VA Medical Center. Due to veterans and PD patients being predominantly men, we could only recruit seven women (four were included in the analysis). Therefore, the potential gender bias was not adequately addressed. In addition, the sample population was primarily Caucasian (three African American subjects).

Many outcome scores within the trial were self-reported surveys, including the FSS, VAFS, and GDS, although the groups were not found to be different at baseline. Furthermore, one year of niacin supplementation may be necessary to find significant differences from placebo; thus, a more extended placebo-controlled study should be performed in the future.

## 5. Conclusions

The findings presented here show that treatment with low-dose daily niacin supplementation compared to placebo resulted in significantly improved motor function outcomes. We have already demonstrated that niacin acts by polarizing the activated microglial cells in the brain and reducing GPR109A expression in white blood cells, thus reducing neuroinflammation. Our findings support the use of low-dose niacin supplementation in PD as an adjunct therapy that can reduce neuroinflammation and improve motor functions in PD patients.

## Figures and Tables

**Table 1:** Demographic and baseline characteristics of the participants (n = 39).

	Treatment Groups		
Characteristics	Placebo (n = 21)	Niacin (n = 18)	*p*-Value
Sex, N (%)			
Men	19 (90.5)	16 (88.9)	1 ^a^
Women	2 (9.5)	2 (11.1)	
Race/ethnicity, N (%)			
Non-Hispanic White	18 (85.7)	18 (100)	0.29 ^a^
Non-Hispanic Black	3 (14.3)	0 (0)	
Veteran status, N (%)			
Veterans	15 (71.4)	10 (55.6)	0.5 ^a^
Non-Veterans	6 (28.6)	8 (44.4)	
Age, mean (SD), y	68.0 (10.7)	68.2 (6.0)	
Duration of PD, mean (SD), y	5.6 (4.2)	6.0 (6.0)	
Age of Onset, mean (SD), y	61.6 (10.9)	63.3 (7.2)	
Disease Stage, N (%)			
Early (H&Y < 2.5)	14 (66.7)	14 (77.8)	0.68 ^a^
Late (H&Y ≥ 2.5)	7 (33.3)	4 (22.2)	
Medications, mg/day			
Sinemet intake, N (%)	20 (95.2)	15 (83.3)	0.5 ^a^
Levodopa dosage, mean (range)	488.9 (300–1000)	480.8 (200–800)	
H&Y staging, mean (range)	2.2 (1.5–4)	1.8 (0.5–4)	
UPDRS III Scores, mean (SD)	22.4 (11.8)	21.3 (15.8)	0.99 ^b^
Rigidity	1.69 (2.23)	1.5 (1.92)	0.99 ^b^
Resting Tremor	4.52 (2.87)	3.11 (2.82)	0.34 ^b^
Bradykinesia	4.5 (4.6)	4 (4.5)	0.98 ^b^
Cognitive flexibility	73.38 (57.11)	79.42 (62.46)	0.99 ^b^
Grip Strength, PSI			
First affected hand	305.09 (105.26)	297.04 (125.68)	0.99 ^b^
Non (or later) affected hand	300.43 (80.49)	284.1 (123.09)	0.98 ^b^
FSS	36.65 (13.02)	40.28 (11.99)	0.76 ^b^
VAFS	5.4 (2.23)	5.17 (2.23)	0.96 ^b^
REM sleep, %	15.19 (12.1)	22.5 (13.36)	0.32 ^b^
GDS	6.2 (5.87)	7.89 (7.31)	0.66 ^b^
Stroop 3 trial	8.13 (6.64)	6.18 (7.92)	0.81 ^b^
Walk and Turn, s	11.47 (3.46)	10.33 (3.16)	0.66 ^b^
Valine, mg/dL	229.33 (51.78)	245.64 (33.42)	0.69 ^b^
Tyrosine, mg/dL	73.93 (14.77)	64.93 (16.28)	0.35 ^b^
Tryptophan, mg/dL	60.87 (11.39)	53.5 (10.81)	0.23 ^b^
Serotonin, mg/dL	89.56 (60.5)	84.21 (68.17)	0.99 ^b^
Phenylalanine, mg/dL	74.47 (12.59)	73.07 (9.74)	0.98 ^b^
Leucine, mg/dL	136.87 (38.75)	143.07 (27.71)	0.95 ^b^
Isoleucine, mg/dL	72.14 (24.2)	73.36 (15)	0.99 ^b^

Values presented are proportions and mean (SD). a—differences were tested by chi-squared test, b—differences in means was test by t-test. UPDRS III–Unified Parkinson’s disease Rating Scale III, H&Y-Hoehn and Yahr Scale for Parkinson’s Disease Staging. Levodopa dosage was determined as mg/day. * Medication information on two subjects in the niacin group was not available.

**Table 2:** Differences in motor and cognitive scores during the randomized trial period (baseline vs. 6MO).

Clinical	Baseline Values, Mean ±SD		6-Month Change (Randomized, Placebo-Controlled Trial)		
Variable, Units	Placebo Group	Niacin Group	Placebo Group Change	Niacin Group Change	Between Group Difference
UPDRS III Scores	22.4 ± 11.8	21.3 ± 15.8	−0.05 (−2.4–2.32)	−1.06 (−3.68–1.57)	1.13 (−10.40–12.65)
Rigidity	1.69 ± 2.23	1.5 ± 1.92	0.14 (−0.14–0.42)	0.03 (−0.26–0.31)	0.19 (−1.47–1.85)
Resting Tremor	4.52 ± 2.87	3.11 ± 2.82	0.05 (−0.54–0.64)	−0.17 (−0.49–0.16)	1.4 (−0.87–3.7)
Bradykinesia	4.5 ± 4.6	4 ± 4.5	0.07 (−0.35–0.49)	−0.39 (−1.42–0.65)	0.5 (−3.15–3.7)
Cognitive flexibility	73.38 ± 57.11	79.42 ± 62.46	−3.04 (−28.04–21.96)	5.44 (−30.78–41.67)	−6.04 (−54.93–42.85)
Grip Strength, PSI					
First affected hand	305.09 ± 105.26	297.04 ± 125.68	5.88 (−14.19–25.94)	−22.56 (−53.54–8.43)	8.05 (−132.3–148.4)
Non (or later) affected hand	300.43 ± 80.49	284.1 ± 123.09	3.73 (−35.11–42.56)	−31.67 (−63.74–0.39)	16.33 (−110–142.7)
FSS	36.65 ± 13.02	40.28 ± 11.99	−0.06 (−2.5–2.37)	1.78 (−5.5–9.05)	−3.63 (−13.79–6.53)
VAFS	5.4 ± 2.23	5.17 ± 2.23	−0.22 (−0.97–0.53)	−0.44 (−1.74–0.85)	0.23 (−1.58–2.05)
REM sleep, %	15.19 ± 12.1	22.5 ± 13.36	−6.26 (−11.05–1.47) *	1.39 (−5.42–8.2)	−7.31 (−17.62–3.0)
GDS	6.2 ± 5.87	7.89 ± 7.31	0.25 (−0.53–1.03)	1.17 (−1.75–4.08)	−1.69 (−7.14–3.76)
Stroop 3 trial	8.13 ± 6.64	6.18 ± 7.92	−0.59 (−3.17–1.99)	−2.19 (−5.93–1.54)	1.96 (−4.16–8.08)
Walk and Turn, s	11.47 ± 3.46	10.33 ± 3.16	0.44 (−1.08–1.96)	0.18 (−0.61–0.96)	1.14 (−1.61–3.88)
Valine, mg/dL	229.33 ± 51.78	245.64 ± 33.42	1.83 (−27.05–30.72)	−0.71 (−35.17–33.74)	−16.31 (−57.55–24.93)
Tyrosine, mg/dL	73.93 ± 14.77	64.93 ± 16.28	8.82 (−2.2–19.84)	−0.5 (−10.82–9.82)	9.01 (−5.75–23.76)
Tryptophan, mg/dL	60.87 ± 11.39	53.5 ± 10.81	7.48 (−0.39–15.34)	−0.07 (−6.1–5.96)	7.37 (−3.13–17.86)
Serotonin, mg/dL	89.56 ± 60.5	84.21 ± 68.17	25.34 (5.79–44.89) *	1.21 (−17.82–20.25)	5.35 (−55.03–65.72)
Phenylalanine, mg/dL	74.47 ± 12.59	73.07 ± 9.74	3.74 (−8.48–15.97)	−1.64 (−8.62–5.33)	1.40 (−9.23–12.02)
Leucine, mg/dL	136.87 ± 38.75	143.07 ± 27.71	9.26 (−15.26–33.77)	−4.64 (−28.45–19.16)	−6.21 (−38.02–25.61)
Isoleucine, mg/dL	72.14 ± 24.2	73.36 ± 15	4.03 (−14.21–22.26)	−4.79 (−19.34–9.77)	−1.21 (−20.89–18.47)

All of the study personnel and patients were blinded to the group assignment. The treatment group assignment code was disclosed at the completion of the study in April 2020. VAFS: Visual analog severity scale, GDS: Geriatric depression scale, FSS: fatigue severity scale. Values presented are Mean ± SD or Mean (95% CI). * *p* < 0.05.

**Table 3:** Comparative differences in motor and cognitive scores during the open-label trial.

Clinical	6-Month Values (Mean ±SD)		6–12 Month Change (Open-Label Niacin Treatment)		
Variable, Units	Placebo Group	Niacin Group	Placebo Group Change	Niacin Group Change	Between Group Difference
UPDRS III Scores	22.4 ± 14	22.3 ± 16.4	4.58 (0.85–8.30) *	4.63 (1.42–7.83) *	0.12 (−12.29–12.53)
Rigidity	1.5 ± 2.1	1.5 ± 1.9	0.75 (−0.01–1.51) *	0.008 (−0.47–0.49)	0.08 (−1.522–1.673)
Resting Tremor	4.5 ± 3.3	3.3 ± 2.7	0.92 (−0.12–1.96)	0.1 (−0.56–0.76)	1.20 (−1.17–3.57)
Bradykinesia	4.4 ± 4.4	4.4 ± 4.9	1.13 (0.25–2.02) *	1.21 (0.36–2.06) *	0.04 (−3.74–3.82)
Cognitive flexibility	76.4 ± 63.1	74 ± 54.7	−15.18 (−40.6–10.24)	4.9 (−27.8–37.6)	2.44 (−47.02–51.91)
Grip Strength, Newtons					
First affected hand	299.2 ± 107.5	319.6 ± 118.2	−23.58 (−79.14–31.98)	−80.3 (−176.2–15.65)	−20.38 (−167.4–126.7)
Non (or later) affected hand	296.7 ± 89.4	315.8 ± 119.4	−11.84 (−56.59–32.92)	−54.72 (−128.8–19.38)	−19.07 (−152.8–114.7)
FSS	36.7 ± 14.2	38.5 ± 11	2.89 (−1.62–7.4)	4.36 (1.59–7.13) *	−1.79 (−11.91–8.34)
VAFS	5.6 + 2.5	5.6 ± 2.2	−0.76 (−1.75–0.24)	−0.39 (−0.92–0.14)	0.008 (−1.88–1.89)
REM sleep, %	6 ± 5.9	6.7 ± 5.9	1.92 (−7.2–11.03)	−4.32 (−10.32–1.68)	0.34 (−11.09–11.77)
GDS	21.5 ±16.3	21.1 ± 11.6	1.08 (−1.12–3.28)	1.37 (−0.5–3.23)	−0.77 (−5.53–3.99)
Stroop 3 trial average	8.7 ± 6.9	8.4 ± 5.6	−0.78 (−5.47–3.91)	−0.78 (−2.85–1.28)	0.35 (−4.9–5.61)
Walk and Turn, s	11 ± 3.2	10.2 ± 3.4	0.75 (−0.53–2.02)	1.72 (0.59–2.84) *	0.87 (−1.95–3.69)
Valine, mg/dL	227.5 ± 45	246.4 ± 29.7	4.29 (−32.51–41.08)	21.07 (−16.01–58.15)	−18.86 (−52.4–14.69)
Tyrosine, mg/dL	65.1 ± 15.5	65.4 ± 14.4	1.83 (−11.66–15.31)	4.57 (−5.33–14.47)	−0.32 (−13.74–13.11)
Tryptophan, mg/dL	53.4 ± 11.6	53.6 ± 10.9	−4.11 (−12.97–4.75)	4.29 (−4.75–13.32)	−0.18 (−10.28–9.92)
Serotonin, mg/dL	64.2 ± 44.5	83 ± 75.2	−18.11 (−37.97–1.75)	−13.36 (−58.77–32.06)	−18.78 (−77.83–40.27)
Phenylalanine, mg/dL	70.7 ± 20.6	74.7 ± 9.8	−0.85 (−12–10.3)	2.43 (−9.35–14.21)	−3.99 (−18.11–10.12)
Leucine, mg/dL	127.6 ± 32.5	147.7 ± 21.7	−1.75 (−29.99–26.5)	14.86 (−9.31–39.02)	−20.1 (−44.43–4.23)
Isoleucine, mg/dL	68.1 ± 20.4	78.1 ± 15.2	1.26 (−14.45–16.97)	8.64 (−4.48–21.77)	−10.03 (−26.28–6.230)

Six to 12 months, all the participants were given 250 mg niacin once a day. All the study personnel and patients were blinded to the group assignment. Treatment group assignment code was disclosed at the completion of the study in April 2020. Values are presented as Mean ± SD or Mean (95% CI). * *p* < 0.05.

**Table 4:** Differences in motor and cognitive scores between baseline and 12-month visit.

	12-Month Values (Mean ±SD)		12- Month Change		
Clinical Variable, Units	Placebo Group	Niacin Group	Placebo Group Change	Niacin Group Change	Between Group Difference
UPDRS III Scores	17.9 ± 9.2	17.7 ± 13.3	4.52 (−0.16–9.21)	3.57 (−1.019–8.16)	0.17 (−10.66–11.01)
Rigidity	0.8 ± 1.5	1.5 ± 1.9	0.90 (−0.05–1.84)	0.04 (−0.43–0.5)	−0.67 (−2.25–0.91)
Resting Tremor	3.6 ± 2.9	3.2 ± 2.7	0.97 (−0.16–2.09)	−0.07 (−0.94–0.81)	0.38 (−2.18–2.94)
Bradykinesia	3.3 ± 2.8	3.2 ± 4.3	1.21 (0.19–2.23) *	0.82 (−0.004–1.65) *	0.12 (−3.34–3.57)
Cognitive flexibility	91.6 ± 72	69.1 ± 55.5	18.22 (−42.79–6.35)	10.35 (−17.7–38.39)	22.52 (−36.72–81.77)
Grip Strength, Newtons					
First affected hand	322.8 ± 91.7	399.9 ± 54.1	−17.71 (−62.17–26.75)	−102.9 (−218.8–13.14)	−77.1 (−186.4–32.22)
Non (or later) affected hand	308.5 ± 84.6	370.5 ± 75.3	−8.12 (−56.34–40.13)	−86.4 (−187.8–15.05)	−61.96 (−181.5–57.59)
FSS	33.8 ± 13.6	34.1 ± 12	2.83 (−2.15–7.81)	6.14 (−1.59–13.86)	−0.32 (−12–11.37)
VAFS	6.4 ± 2.2	6 ± 2.5	−0.98 (−2.2–0.25)	−0.83 (−2.16–0.49)	0.38 (−1.84–2.59)
REM sleep, %	4.9 ± 4.9	5.4 ± 5.3	−4.34 (−11.45–2.76)	−2.93 (−11.11–5.26)	−5.9 (−20.57–8.78)
GDS	19.5 ± 16.8	25.4 ± 14.1	1.33 (−1.01–3.66)	2.53 (−1.56–6.62)	−0.48 (−5.24–4.28)
Stroop 3 trial average	9.5 ± 7.5	9.2 ± 6.3	−1.37 (−5.41–2.67)	−2.98 (−7.73–1.78)	0.35 (−6.45–7.14)
Walk and Turn, s	10.3 ± 2.8	8.4 ± 1.7	1.19 (−0.38–2.76)	1.89 (0.55–3.23) *	1.84 (−0.39–4.07)
Valine, mg/dL	223.2 ± 37.7	225.3 ± 37.9	6.12 (−29.16–41.4)	20.36 (−17.62–58.33)	−2.07 (−38.5–34.35)
Tyrosine, mg/dL	63.3 ± 12.1	60.9 ± 9.2	10.65 (1.19–20.1) *	4.07 (−8.28–16.42)	2.43 (−7.98–12.84)
Tryptophan, mg/dL	57.5 ± 9.3	49.3 ± 10.8	3.37 (−5.21–11.94)	4.21 (−3.69–12.12)	8.21 (−1.54–17.97)
Serotonin, mg/dL	82.3 ± 31.6	96.4 ± 56.3	7.23 (−24.06–38.51)	−12.14 (−49.59–25.31)	−14.02 (−58.56–30.51)
Phenylalanine, mg/dL	71.6 ± 11	72.3 ± 13.5	2.9 (−6.82–12.61)	0.79 (−10.03–11.6)	−0.71 (−12.62–11.19)
Leucine, mg/dL	129.4 ± 24.6	132.9 ± 28.9	7.51 (−18.63–33.65)	10.21 (−21.1–41.53)	−3.5 (−29.45–22.45)
Isoleucine, mg/dL	66.9 ± 11.2	69.5 ± 16	5.29 (−8.82–19.39)	3.86 (−10.4–18.12)	−2.64 (−16.11–10.82)

The study personnel were blinded to the group assignment; the randomization code was revealed at the completion of the study. Values presented are Mean ± SD or Mean (95% CI). * *p* < 0.05.

## Data Availability

Upon reasonable request, de-identified individual participant data will be available beginning 9 months and ending 36 months following the publication of this article.

## Supplementary Materials

The following are available online at https://www.mdpi.com/article/10.3390/biomedicines9121881/s1, Table S1: Mean values for clinical variables measured for the two treatment groups at the baseline, Table S2: Comparative differences in motor and cognitive scores during randomized period (baseline vs 6months), open label trial (6 vs 12months) and baseline to study end (baseline vs 12 months).
