# Supplementary material for: Low-Dose Niacin Supplementation Improves Motor Function in US Veterans with Parkinson’s Disease: A Single-Center, Randomized, Placebo-Controlled Trial
Source: Biomedicines. 2021 Dec 10;9(12):1881. doi: 10.3390/biomedicines9121881 (PMC8698595; doi:10.3390/biomedicines9121881)
Supplement: Supplementary file 1 [file biomedicines-09-01881-s001.zip › biomedicines-1453528-supplementary.pdf]

Supplmentary Table S1: Mean values for clinical variables measured for the two treatment groups at the baseline

	Baseline Values, Mean (SD)	
Variable, units	Placebo Group	Niacin Group
UPDRS III Scores	22.4 ± 11.8	21.3 ± 15.8
Rigidity	1.69 ± 2.23	1.5 ± 1.92
Resting Tremor	4.52 ± 2.87	3.11 ± 2.82
Bradykinesia	4.5 ± 4.6	4 ± 4.5
Cognitive flexibility	73.38 ± 57.11	79.42 ± 62.46
Grip Strength, PSI		
First affected hand	305.09 ± 105.26	297.04 ± 125.68
Non (or later) affected hand	300.43 ± 80.49	284.1 ± 123.09
FSS	36.65 ± 13.02	40.28 ± 11.99
VAFS	5.4 ± 2.23	5.17 ± 2.23
REM sleep, %	15.19 ± 12.1	22.5 ± 13.36
GDS	6.2 ± 5.87	7.89 ± 7.31
Stroop 3 trial	8.13 ± 6.64	6.18 ± 7.92
Walk and Turn, s	11.47 ± 3.46	10.33 ± 3.16
Valine, mg/dL	229.33 ± 51.78	245.64 ± 33.42
Tyrosine, mg/dL	73.93 ± 14.77	64.93 ± 16.28
Tryptophan, mg/dL	60.87 ± 11.39	53.5 ± 10.81
Serotonin, mg/dL	89.56 ± 60.5	84.21 ± 68.17
Phenylalanine, mg/dL	74.47 ± 12.59	73.07 ± 9.74
Leucine, mg/dL	136.87 ± 38.75	143.07 ± 27.71
Isoleucine, mg/dL	72.14 ± 24.2	73.36 ± 15

Supplementary Table S2: Comparative differences in motor and cognitive scores during randomized period (baseline vs 6months), open label trial (6 vs 12months) and baseline to study end (baseline vs 12 months) .

	baseline vs 6-month change (Randomized, placebo-controlled trial)		6 vs 12 month change (open-label niacin treatment)		baseline vs 12- month change	
Clinical Variable, units	Placebo Group Change	Niacin Group Change	Placebo Group Change	Niacin Group Change	Placebo Group Change	Niacin Group Change
UPDRS III Scores	-0.05 (-2.4-2.32)	-1.06 (-3.68-1.57)	4.58 (0.85-8.30)*	4.63 (1.42-7.83)*	4.52 (-0.16-9.21)	3.57 (-1.019-8.16)
Rigidity	0.14 (-0.14-0.42)	0.03 (-0.26-0.31)	0.75 (-0.01-1.51)*	0.008 (-0.47-0.49)	0.90 (-0.05-1.84)	0.04 (-0.43-0.5)
Resting Tremor	0.05 (-0.54-0.64)	-0.17 (-0.49-0.16)	0.92 (-0.12-1.96)	0.1 (-0.56-0.76)	0.97 (-0.16-2.09)	-0.07 (-0.94-0.81)
Bradykinesia	0.07 (-0.35-0.49)	-0.39 (-1.42-0.65)	1.13 (0.25-2.02)*	1.21 (0.36-2.06)*	1.21 (0.19-2.23)*	0.82 (-0.004-1.65)*
Cognitive flexibility	-3.04 (-28.04-21.96)	5.44 (-30.78-41.67)	-15.18 (-40.6-10.24)	4.9 (-27.8-37.6)	18.22 (-42.79-6.35)	10.35 (-17.7-38.39)
Grip Strength, PSI						
First affected hand	5.88 (-14.19-25.94)	-22.56 (-53.54-8.43)	-23.58 (-79.14-31.98)	-80.3 (-176.2-15.65)	-17.71 (-62.17-26.75)	-102.9 (-218.8-13.14)
Non (or later) affected hand	3.73 (-35.11-42.56)	-31.67 (-63.74-0.39)	-11.84 (-56.59-32.92)	-54.72 (-128.8-19.38)	-8.12 (-56.34-40.13)	-86.4 (-187.8-15.05)
FSS	-0.06 (-2.5-2.37)	1.78 (-5.5-9.05)	2.89 (-1.62-7.4)	4.36 (1.59-7.13)*	2.83 (-2.15-7.81)	6.14 (-1.59-13.86)
VAFS	-0.22 (-0.97-0.53)	-0.44 (-1.74-0.85)	-0.76 (-1.75-0.24)	-0.39 (-0.92-0.14)	-0.98 (-2.2-0.25)	-0.83 (-2.16-0.49)
REM sleep, %	-6.26 (-11.05-1.47)*	1.39 (-5.42-8.2)	1.92 (-7.2-11.03)	-4.32 (-10.32-1.68)	-4.34 (-11.45-2.76)	-2.93 (-11.11-5.26)
GDS	0.25 (-0.53-1.03)	1.17 (-1.75-4.08)	1.08 (-1.12-3.28)	1.37 (-0.5-3.23)	1.33 (-1.01-3.66)	2.53 (-1.56-6.62)
Stroop 3 trial	-0.59 (-3.17-1.99)	-2.19 (-5.93-1.54)	-0.78 (-5.47-3.91)	-0.78 (-2.85-1.28)	-1.37 (-5.41-2.67)	-2.98 (-7.73-1.78)
Walk and Turn, s	0.44 (-1.08-1.96)	0.18 (-0.61-0.96)	0.75 (-0.53-2.02)	1.72 (0.59-2.84)*	1.19 (-0.38-2.76)	1.89 (0.55-3.23)*
Valine, mg/dL	1.83 (-27.05-30.72)	-0.71 (-35.17-33.74)	4.29 (-32.51-41.08)	21.07 (-16.01-58.15)	6.12 (-29.16-41.4)	20.36 (-17.62-58.33)
Tyrosine, mg/dL	8.82 (-2.2-19.84)	-0.5 (-10.82-9.82)	1.83 (-11.66-15.31)	4.57 (-5.33-14.47)	10.65 (1.19-20.1)*	4.07 (-8.28-16.42)
Tryptophan, mg/dL	7.48 (-0.39-15.34)	-0.07 (-6.1-5.96)	-4.11 (-12.97-4.75)	4.29 (-4.75-13.32)	3.37 (-5.21-11.94)	4.21 (-3.69-12.12)
Serotonin, mg/dL	25.34 (5.79-44.89)*	1.21 (-17.82-20.25)	-18.11 (-37.97-1.75)	-13.36 (-58.77-32.06)	7.23 (-24.06-38.51)	-12.14 (-49.59-25.31)
Phenylalanine, mg/dL	3.74 (-8.48-15.97)	-1.64 (-8.62-5.33)	-0.85 (-12-10.3)	2.43 (-9.35-14.21)	2.9 (-6.82-12.61)	0.79 (-10.03-11.6)
Leucine, mg/dL	9.26 (-15.26-33.77)	-4.64 (-28.45-19.16)	-1.75 (-29.99-26.5)	14.86 (-9.31-39.02)	7.51 (-18.63-33.65)	10.21 (-21.1-41.53)
Isoleucine, mg/dL	4.03 (-14.21-22.26)	-4.79 (-19.34-9.77)	1.26 (-14.45-16.97)	8.64 (-4.48-21.77)	5.29 (-8.82-19.39)	3.86 (-10.4-18.12)

Six-months to 12-month time all the participants were given 250mg niacin once a day. All the study personnel and patients were blinded to the group assignment. Treatment group assignment code was disclosed at the completion of the study in April 2020. Values presented are Mean (95% CI). *p<0.05
